# Integrative Transcriptomic Analysis and Single-Cell Validation Identify a Six-Hub-Gene Signature Converging on Inflammatory Signaling in Osteoarthritis

**DOI:** 10.3390/genes17060696

**Published:** 2026-06-15

**Authors:** Xueya Lv, Yang Yu, Jiawen Fan, Lianjiang Guo, Xiang Zhu, Xingye Li

**Affiliations:** 1 Department of Orthopedics Medicine, Beijing Jishuitan Hospital, Capital Medical University, Beijing 100035, China; lxy123lina@163.com; 2 Department of Orthopedics, Army No. 82 Group Military Hospital, Baoding 071000, China; yuyangfresh@126.com (Y.Y.); 13703285699@139.com (J.F.); 18617786122@163.com (L.G.); 3 Department of Oncology, Army No. 82 Group Military Hospital, Baoding 071000, China; 4 Department of Spine Surgery, Beijing Jishuitan Hospital, Capital Medical University, Beijing 100035, China

**Keywords:** osteoarthritis, cartilage, ECM remodeling, ER stress, inflammatory signaling, diagnostic model

## Abstract

Background: Osteoarthritis (OA) is a heterogeneous joint disease characterized by cartilage degeneration. The interplay between extracellular matrix (ECM) remodeling, endoplasmic reticulum (ER) stress, and inflammatory signaling in OA pathogenesis remains incompletely understood. This study aimed to identify robust diagnostic biomarkers and explore the mechanistic convergence of key genes in OA cartilage through an integrated transcriptomic framework. Methods: Three independent cartilage transcriptomic datasets (GSE285234, GSE287861, GSE289464) were integrated after ComBat batch correction. Differentially expressed genes (DEGs) were identified using limma, followed by ORA and GSEA for functional enrichment. LASSO logistic regression identified hub genes for a diagnostic model and nomogram, validated by leave-one-out cross-validation (LOOCV). Consensus clustering stratified OA samples into molecular subtypes. Single-cell RNA-sequencing (scRNA-seq) data (GSE169454, GSE220243) were used to validate cell-type-specific expression. Virtual gene knockout (scTenifoldKnk) and pathway analysis inferred downstream functional consequences. Results: Fifty-eight DEGs (predominantly downregulated) were enriched in ECM and ER protein processing pathways. Six hub genes (EIF2S1, GANAB, STT3A, XBP1, MGP, PMP22) showed robust selection stability. The diagnostic model achieved a LOOCV AUC of 0.769, a well-calibrated nomogram, and superior net benefit. Unsupervised clustering revealed two OA subtypes with divergent unfolded protein response (UPR) and TGF-β pathway activities. scRNA-seq confirmed hub gene expression in chondrocytes and other joint microenvironment cells. Notably, virtual knockout of five hub genes convergently perturbed IL-17, NF-κB, and chemokine signaling pathways. Conclusions: This study identified and validated a six-gene signature reflecting ECM-ER-inflammatory crosstalk in OA cartilage. The convergent perturbation of inflammatory pathways by functionally distinct hub genes reveals a mechanistic core that may serve as a diagnostic panel and a platform for targeted therapeutic investigation in OA.

## 1. Introduction

Osteoarthritis (OA) is the most prevalent form of arthritis and a leading cause of chronic disability worldwide, imposing a substantial socioeconomic burden on aging populations [1]. The disease is characterized by the progressive degeneration of articular cartilage, accompanied by synovial inflammation, subchondral bone remodeling, and osteophyte formation. Notably, OA is increasingly recognized as a whole-joint disease, involving meniscal degeneration, ligamentous changes, and biomechanical alterations of the infrapatellar fat pad [2], which collectively contribute to disease progression. Despite its high prevalence, the molecular pathogenesis of OA remains incompletely elucidated, and currently, no disease-modifying pharmacological interventions are available to halt or reverse structural disease progression [3]. The clinical management of OA is challenged by its substantial heterogeneity, which manifests as variable rates of progression and differential responses to therapy, underscoring the urgent need for molecular stratification tools and mechanistically anchored therapeutic targets.

Transcriptomic profiling has emerged as a powerful approach to dissect the molecular landscape of OA cartilage. Multiple microarray and RNA-sequencing studies have identified thousands of differentially expressed genes (DEGs) between OA and healthy cartilage, revealing perturbations in extracellular matrix (ECM) homeostasis, inflammatory cascades, chondrocyte hypertrophy, and metabolic adaptation [4]. However, individual studies often suffer from limited sample sizes, platform-specific biases, and cohort-specific confounders, which collectively reduce statistical power and hinder the reproducibility of findings. The integration of multiple independent datasets through robust batch-effect correction and integrative analytical frameworks offers a solution to these limitations, enabling the identification of reproducible and biologically meaningful gene signatures [5]. We focused specifically on cartilage because it constitutes the primary tissue undergoing degeneration in OA, its transcriptomic alterations are among the most extensively profiled, and integrating multiple cartilage-specific datasets provides a focused window into the core molecular pathology.

Beyond the identification of diagnostic biomarkers, a deeper functional understanding of how dysregulated genes mechanistically contribute to OA pathogenesis is essential. The endoplasmic reticulum (ER) has garnered increasing attention in OA research because chondrocytes, as highly secretory cells responsible for synthesizing and maintaining the abundant cartilage ECM, are particularly vulnerable to ER stress [6]. The unfolded protein response (UPR), an adaptive signaling network activated by ER stress, is implicated in chondrocyte survival, matrix catabolism, and inflammatory signaling [7]. Concurrently, chronic low-grade inflammation, mediated by cytokines such as IL-1β, TNF-α, and IL-17, originates in part from the synovial membrane and the infrapatellar fat pad, which secrete pro-inflammatory factors into the joint microenvironment, driving catabolic enzyme expression and perpetuating cartilage degradation [8]. The functional intersection between ECM dysregulation, ER stress, and inflammatory signaling represents a critical frontier for understanding OA pathophysiology, yet the specific molecular nodes linking these processes remain poorly defined.

The advent of single-cell RNA-sequencing (scRNA-seq) has further transformed the study of complex tissues by resolving cellular heterogeneity that is masked in bulk transcriptomic analyses [9]. Recent single-cell atlases of OA cartilage have revealed a diverse cellular ecosystem comprising multiple chondrocyte subtypes, as well as immune and stromal cells that collectively shape the joint microenvironment [10]. Leveraging scRNA-seq to validate and refine the cell-type resolution of bulk-derived gene signatures provides a powerful strategy to bridge population-level transcriptomic findings with cellular mechanism.

In this study, we aimed to construct a comprehensive analytical pipeline integrating multi-dataset bulk transcriptomics, stringent machine learning-based feature selection, diagnostic model construction, molecular subtyping, single-cell validation, and in silico functional perturbation. Through this framework, we sought to identify a robust hub gene signature for OA, assess its diagnostic performance, and uncover the mechanistic convergence of these genes on key pathological pathways—thereby providing a multi-dimensional view of the molecular dysregulation underlying osteoarthritis.

## 2. Materials and Methods

### 2.1. Data Acquisition and Preprocessing

Three OA cartilage transcriptomic datasets were selected from GEO based on the following criteria: (1) human articular cartilage samples; (2) inclusion of both OA and non-OA control groups; and (3) availability of raw or preprocessed expression matrices. The resulting integrated dataset comprised GSE285234, GSE287861, and GSE289464, which were retrieved from the Gene Expression Omnibus (GEO) database (Supplementary Table S2) [11,12,13]. The integrated dataset comprised 28 samples (14 OA and 14 control). Raw expression matrices were individually background-corrected, quantile-normalized, and log2-transformed. Probes were mapped to gene symbols, and for genes represented by multiple probes, the probe with the highest mean expression was retained. Genes retained in all three datasets after preprocessing were used for downstream integration. All preprocessing steps were performed using R (version 4.4.2).

### 2.2. Batch Effect Correction and Differential Expression Analysis

The three datasets were merged by common genes using dplyr and tidyr. Batch effects were corrected using the ComBat algorithm from the sva R package [14]. Principal component analysis (PCA) was performed before and after correction to visualize the removal of technical variation. Spearman correlation analysis of the corrected expression matrix was used to further assess inter-sample relationships. Differentially expressed genes (DEGs) between OA and control samples were identified using the limma package with a *p* < 0.05 threshold [15]. The choice of normalization and differential expression approach was informed by comparative benchmarking studies [16]. Volcano plots and heatmaps were generated with ggplot2, ggrepel, pheatmap, and RColorBrewer. All analyses were conducted in R. Additional R packages used in this study included: VennDiagram, patchwork, ggpubr for visualization; clusterProfiler, enrichplot, org.Hs.eg.db, msigdbr for functional enrichment; glmnet, pROC, caret, rms, rmda, reshape2 for machine learning and modeling; ConsensusClusterPlus, GSVA, GSEABase for clustering; Seurat, SingleR, celldex, AUCell, CellChat, monocle3, viridis for single-cell analysis; and scTenifoldKnk, igraph, Matrix for virtual gene knockout.

### 2.3. Functional Enrichment Analysis

Over-representation analysis (ORA) of significant DEGs was performed using clusterProfiler (version 4.20.0) for Gene Ontology (GO) Cellular Component (CC), Molecular Function (MF), and Kyoto Encyclopedia of Genes and Genomes (KEGG) pathways [17]. Benjamini–Hochberg correction was applied for multiple testing (adjusted *p* < 0.05). Gene Set Enrichment Analysis (GSEA) was performed on genes pre-ranked by log2 fold change using the fgsea package (version 1.38.0) against the Hallmark and KEGG gene set collections [18]. Candidate genes were selected by intersecting significant DEGs with core enrichment genes from the top pathways.

### 2.4. Identification of Hub Genes by Lasso Regression

Least absolute shrinkage and selection operator (LASSO) logistic regression was applied to the 15 candidate genes using the glmnet R package [19]. Ten-fold cross-validation determined the optimal penalty parameter λ. The minimum deviance λ (λ.min) was used to select hub genes. Coefficient stability was assessed by 100 bootstrap resampling iterations, and selection frequency was recorded for each gene.

### 2.5. Diagnostic Model Construction and Validation

A multivariable logistic regression model incorporating the six hub genes was constructed. Model performance was rigorously evaluated using leave-one-out cross-validation (LOOCV), with the area under the receiver operating characteristic curve (AUC), sensitivity, specificity, and accuracy computed. A nomogram was built using the rms R package. Calibration was assessed with 1000 bootstrap replicates, and clinical net benefit was evaluated by decision curve analysis [20].

### 2.6. Consensus Clustering of OA Samples

Unsupervised consensus clustering was performed on the 14 OA samples using the expression profiles of the six hub genes with the ConsensusClusterPlus R package [21]. The optimal cluster number k was determined by the cumulative distribution function (CDF) and delta area plots. Gene set variation analysis (GSVA) was performed using the GSVA package to compare Hallmark pathway activities between OA and control samples, and between identified OA subtypes [22].

### 2.7. Single-Cell RNA-Sequencing Analysis

Two independent scRNA-seq datasets of human OA cartilage (GSE169454 and GSE220243) were analyzed (Supplementary Table S2) [23,24]. Each dataset was independently processed using Seurat v4, including quality control where cells were filtered to retain those expressing 500–5000 genes, with <15% mitochondrial reads and <25% ribosomal reads, SCTransform normalization, PCA dimensionality reduction, Uniform Manifold Approximation and Projection (UMAP) visualization, and shared nearest neighbor (SNN) graph-based clustering [25]. Automated cell-type annotation was performed using SingleR with the Human Primary Cell Atlas as the reference [26]. The expression patterns of the six hub genes were projected onto UMAP embeddings and summarized across identified cell types using dot plots.

### 2.8. Virtual Gene Knockout and Pathway Analysis

In silico knockout of each hub gene was performed on OA chondrocytes from the GSE169454 dataset using the scTenifoldKnk algorithm [27]. This method constructs a virtual knockout by comparing the regulatory network of wild-type cells with a perturbed network derived by setting the target gene’s expression to zero. Differentially expressed genes upon virtual knockout were subjected to GSEA against KEGG pathways, with significantly enriched pathways (adjusted *p* < 0.1) reported.

## 3. Results

### 3.1. Data Integration, Batch Effect Correction, and Identification of Differentially Expressed Genes in Osteoarthritis

**Figure 1:**
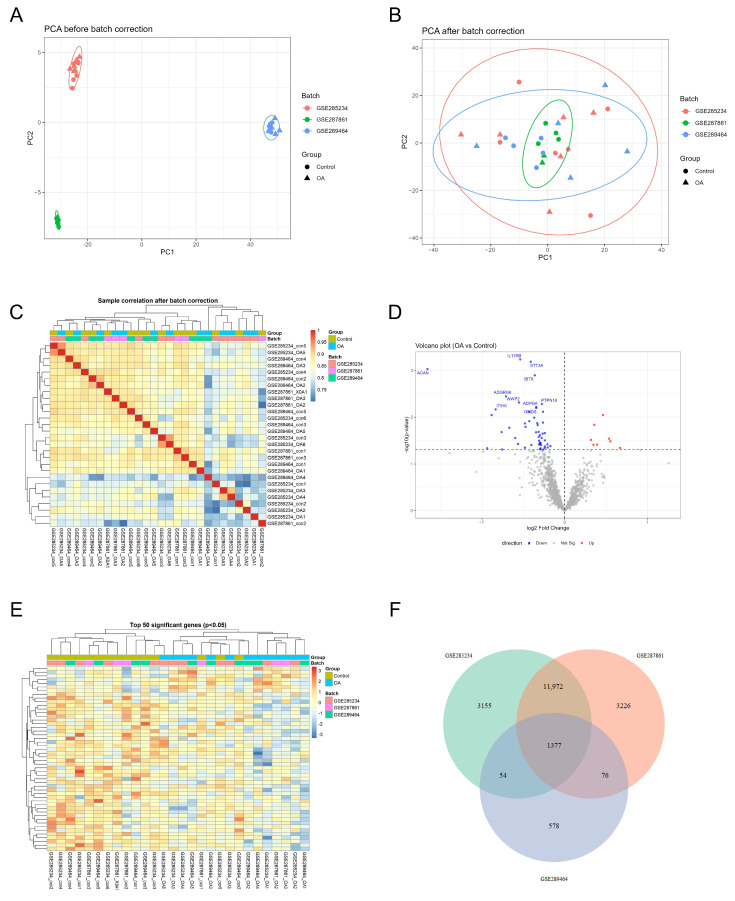
Data integration and identification of DEGs in OA. (**A**) PCA of all samples before batch correction, colored by dataset and shaped by disease status. Samples clustered by dataset origin, indicating severe batch effects. (**B**) PCA after ComBat correction showing improved mixing and emerging separation between OA and control samples. (**C**) Spearman correlation heatmap of the batch-corrected matrix, annotated with dataset and group. (**D**) Volcano plot of differential expression (OA vs. control). Red and blue indicate significantly upregulated and downregulated genes, respectively. The top 10 genes by *p*-value are labeled. (**E**) Heatmap of the top 50 significant genes (row-wise z-score), with hierarchical clustering of samples and genes. (**F**) Venn diagram showing gene overlap across the three datasets.

Three independent OA cartilage transcriptomic datasets (28 samples total; 14 OA, 14 control) were merged by intersecting gene symbols. Principal component analysis (PCA) of the uncorrected matrix revealed severe batch effects, with samples clustering by dataset origin rather than disease status (Figure 1A). After ComBat batch correction, PCA showed markedly improved mixing across datasets and a discernible separation between OA and control samples (Figure 1B). Spearman correlation analysis further confirmed the correction quality (Figure 1C).

Differential expression analysis (limma) identified 58 significantly dysregulated genes (*p* < 0.05) (Supplementary Table S1), of which 50 were downregulated and only 8 upregulated in OA, indicating a predominant transcriptional repression landscape. The volcano plot highlights the top 10 genes by *p*-value (Figure 1D). A heatmap of the top 50 significant genes showed clear separation between OA and control samples (Figure 1E), and a Venn diagram confirmed substantial gene overlap across the three datasets (Figure 1F).

### 3.2. Functional Enrichment Analysis and Candidate Gene Selection

**Figure 2:**
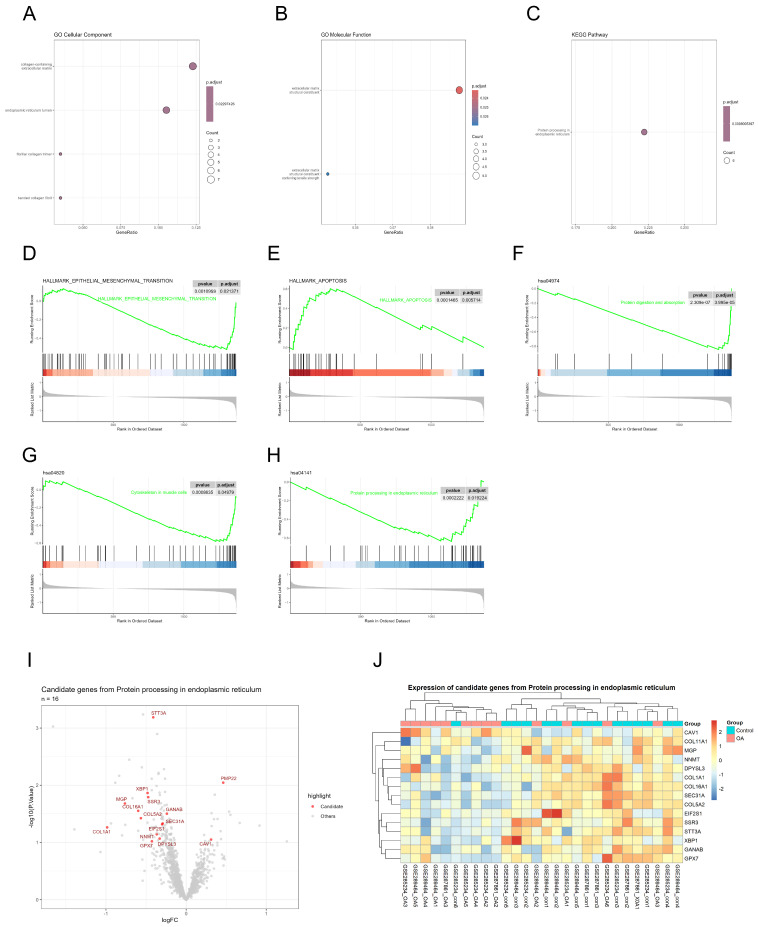
Functional enrichment and candidate gene selection. (**A**–**C**) Dot plots of significantly enriched GO-CC (**A**), GO-MF (**B**), and KEGG pathways (**C**). Dot size indicates gene count; color denotes adjusted *p*-value. (**D**–**H**) GSEA enrichment plots for selected Hallmark (**D**,**E**) and KEGG (**F**–**H**) pathways. NES, normalized enrichment score. (**I**) Volcano plot highlighting the 15 candidate genes in red. (**J**) Heatmap of the 15 candidate genes across all samples (row-wise z-score), with hierarchical clustering.

Over-representation analysis (ORA) of the 58 DEGs revealed prominent enrichment in extracellular matrix (ECM) and endoplasmic reticulum (ER)-related terms. GO-CC terms included “collagen-containing extracellular matrix,” “endoplasmic reticulum lumen,” and “fibrillar collagen trimer” (Figure 2A). GO-MF was dominated by “extracellular matrix structural constituent” (Figure 2B). KEGG pathway analysis highlighted “Protein processing in endoplasmic reticulum” (Figure 2C).

Gene set enrichment analysis (GSEA) independently corroborated these findings: Epithelial–Mesenchymal Transition (EMT) was positively enriched (Figure 2D), while “Apoptosis” showed a trend toward negative enrichment (Figure 2E). Among KEGG pathways, “Protein processing in endoplasmic reticulum” was upregulated, whereas “Protein digestion and absorption” and “Cytoskeleton in muscle cells” were downregulated (Figure 2F–H). Integrating these convergent themes with the DEG list, we defined 15 candidate genes: EIF2S1, GANAB, SEC31A, SSR3, STT3A, XBP1, CAV1, COL16A1, COL1A1, COL5A2, DPYSL3, GPX7, MGP, NNMT, and PMP22 (Figure 2I). Their expression patterns clearly separated OA from control specimens (Figure 2J).

### 3.3. Identification of Hub Genes via Lasso and Stability Assessment

**Figure 3:**
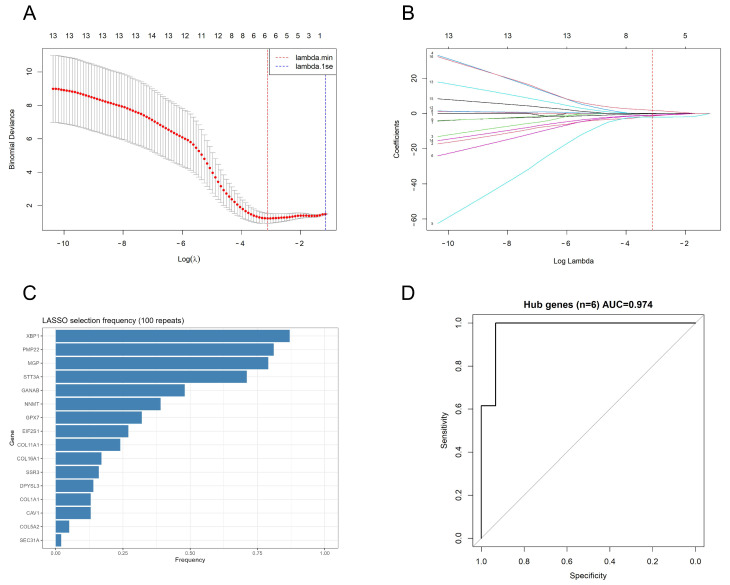
LASSO-based hub gene selection and internal discriminant performance. (**A**) 10-fold cross-validation curve for LASSO regression. The left dotted line indicates the λ value at minimum deviance (λ.min), at which six hub genes were retained. (**B**) Coefficient paths of the 15 candidate genes as a function of log(λ). The dotted line marks λ.min; genes with non-zero coefficients at λ.min are the selected hub genes. (**C**) BAR plot of LASSO selection frequencies from 100 bootstrap replications, showing high stability of the six hub genes. (**D**) ROC curve of the logistic regression model with the six hub genes (AUC = 0.974).

LASSO logistic regression with 10-fold cross-validation was applied to the 15 candidate genes. The cross-validation deviance curve retained six hub genes at λ.min: EIF2S1, GANAB, STT3A, XBP1, MGP, and PMP22 (Figure 3A). Coefficient paths showed that non-informative genes were progressively shrunk to zero, while these six maintained non-zero contributions (Figure 3B). Bootstrap resampling (100 iterations) confirmed their high selection stability (frequencies approaching 1.0), in contrast to the infrequent selection of the remaining candidates (Figure 3C). An exploratory logistic regression model with all six hub genes yielded an AUC of 0.974 (Figure 3D), indicating excellent internal discrimination.

### 3.4. Diagnostic Model Construction and Internal Validation

**Figure 4:**
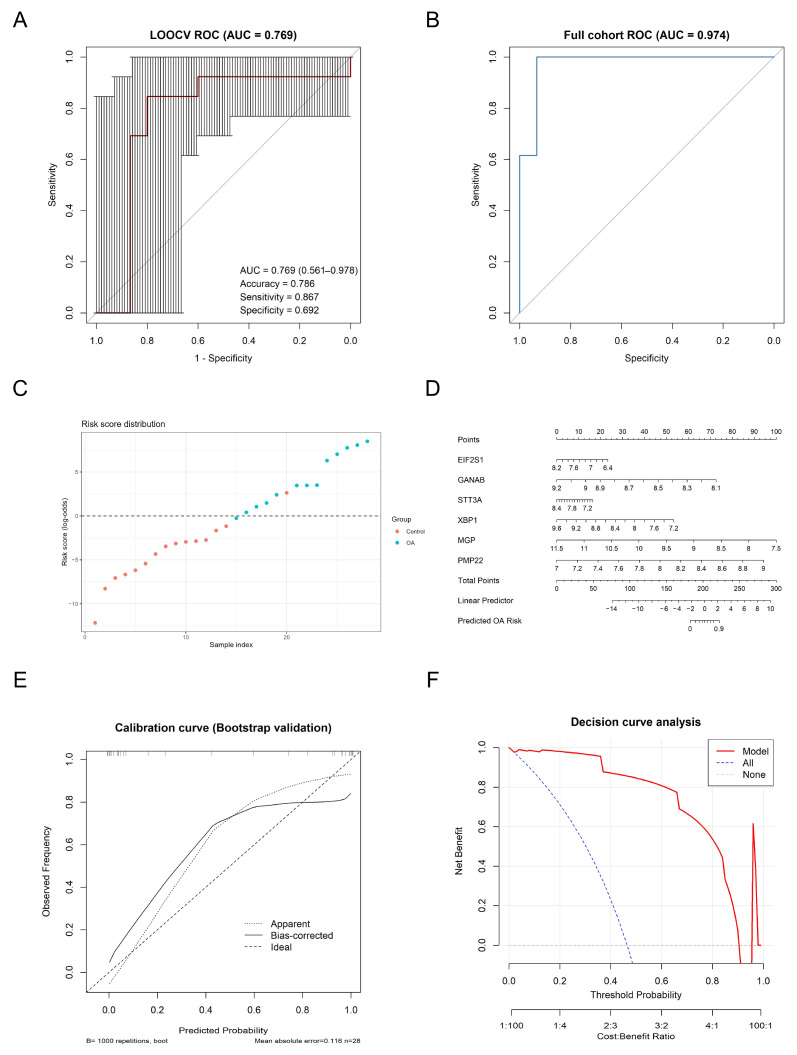
Diagnostic model construction and internal validation. (**A**) ROC curve from LOOCV (AUC = 0.769), with 95% CI and performance metrics at the 0.5 threshold. (**B**) Apparent ROC curve from the full-dataset model (AUC = 0.974). (**C**) Risk score distribution for OA and control samples. (**D**) Nomogram based on the multivariable logistic regression model. (**E**) Calibration curve with 1000 bootstrap replicates. (**F**) Decision curve analysis showing net benefit across threshold probabilities.

A multivariable logistic regression model was constructed using the six hub genes. Leave-one-out cross-validation (LOOCV) estimated generalization performance, yielding an AUC of 0.769 (2000-bootstrap 95% CI excluding 0.5; Figure 4A). Accuracy, sensitivity, and specificity at the 0.5 probability threshold are reported in the legend of Figure 4. In contrast, the apparent ROC curve from the full-dataset model achieved an AUC of 0.974 (Figure 4B), illustrating the optimistic bias of training-set evaluation. The risk score distribution showed higher scores in OA samples with limited overlap (Figure 4C). A nomogram was constructed for clinical translation (Figure 4D), and the calibration curve (1000 bootstrap resamples) demonstrated excellent agreement with the ideal reference line (Figure 4E). Decision curve analysis revealed superior net benefit of the hub gene model compared to “treat all” or “treat none” strategies across a wide range of threshold probabilities (Figure 4F).

### 3.5. Molecular Subtyping of Oa by Consensus Clustering

**Figure 5:**
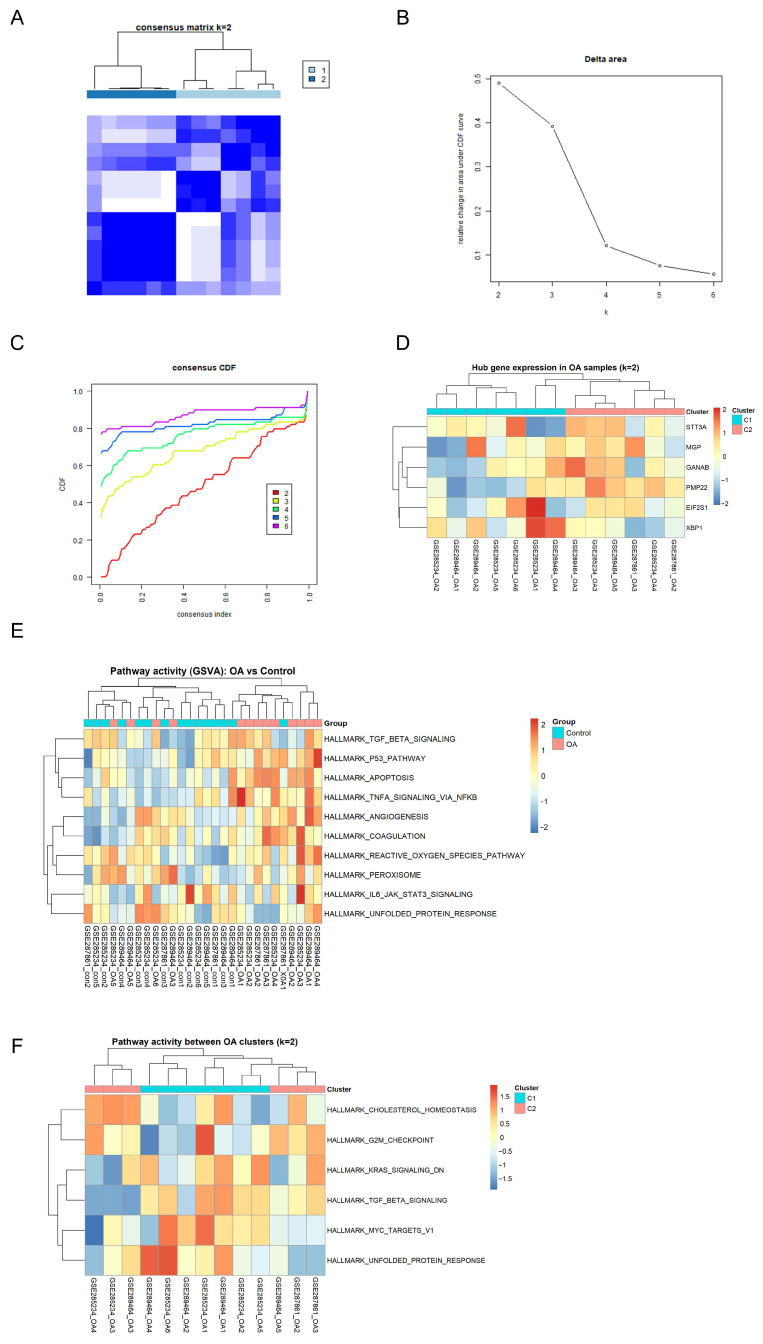
Consensus clustering and pathway activity comparison of OA subtypes. (**A**) Consensus matrix for k = 2. Consensus values range from 0 (blue, never clustered together) to 1 (white, always clustered together). (**B**) Delta area plot supporting k = 2 as the optimal cluster number. (**C**) CDF curves for k = 2 to 6. (**D**) Heatmap of the six hub genes in OA samples, with rows z-score normalized, sorted by subtype (C1 and C2). (**E**) GSVA heatmap of differential Hallmark pathways between OA and control samples. (**F**) GSVA heatmap of differential Hallmark pathways between C1 and C2 subtypes.

As an exploratory analysis, unsupervised consensus clustering of the 14 OA samples based on hub gene expression identified k = 2 as the optimal number of clusters (Figure 5A–C). OA samples were stratified into subtypes C1 and C2 with distinct hub gene expression patterns (Figure 5D).

GSVA revealed significantly dysregulated Hallmark pathways in OA versus control cartilage, including inflammatory signaling (TNFA_SIGNALING_VIA_NFKB, IL6_JAK_STAT3_SIGNALING), ECM remodeling (TGF_BETA_SIGNALING), and cellular stress (UNFOLDED_PROTEIN_RESPONSE, APOPTOSIS) (Figure 5E). Comparison between C1 and C2 further identified differentially active pathways (Figure 5F). C2 exhibited elevated UNFOLDED_PROTEIN_RESPONSE, CHOLESTEROL_HOMEOSTASIS, and TGF_BETA_SIGNALING, while C1 showed higher MYC_TARGETS_V1, G2M_CHECKPOINT, and KRAS_SIGNALING_DN activity. This functional divergence links the ER-resident hub genes (EIF2S1, GANAB, STT3A, XBP1) to a pronounced ER stress and pro-fibrotic phenotype in C2, while C1 displays biosynthetic pathway enrichment.

### 3.6. Single-Cell Transcriptomic Profiling of Hub Genes

**Figure 6:**
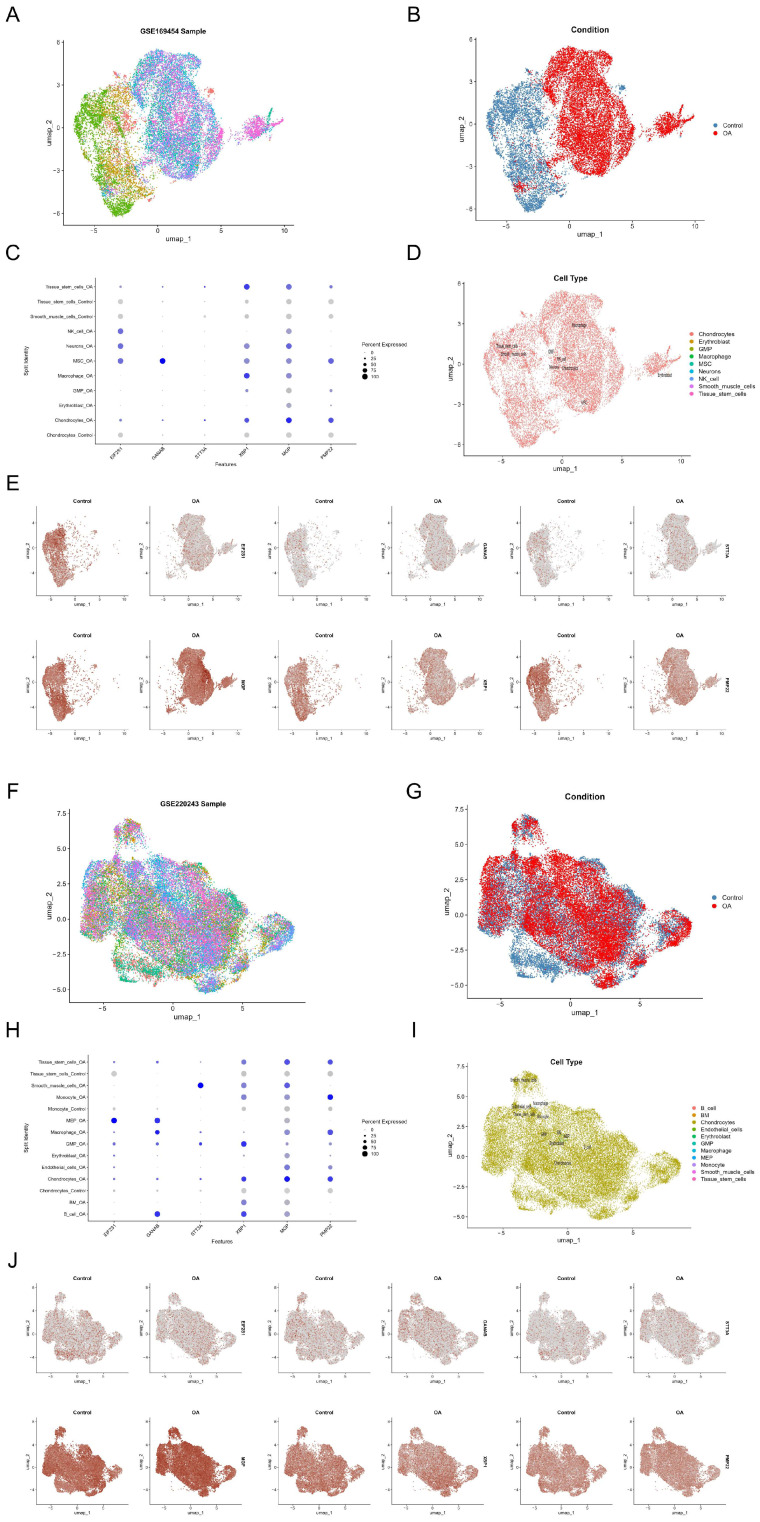
Single-cell transcriptomic profiling of the six hub genes across two independent OA cohorts. (**A**,**F**) UMAP visualizations of GSE169454 and GSE220243, colored by sample identity. (**B**,**G**) UMAP colored by disease condition (red, OA; blue, control). (**C**,**H**) Feature plots of the six hub genes (EIF2S1, GANAB, STT3A, XBP1, MGP, PMP22) on UMAP embeddings, with color gradient from gray (low expression) to blue (high expression). (**D**,**I**) UMAP colored by SingleR-annotated cell types. (**E**,**J**) Dot plots comparing hub gene expression between OA and control across cell types. Dot size: percentage of expressing cells; color intensity: average expression.

To dissect cell-type-specific expression of the six hub genes, two independent scRNA-seq datasets of OA cartilage were analyzed: GSE169454 (7 samples) and GSE220243 (12 samples). Each dataset was processed separately using Seurat, including quality filtering, SCTransform normalization, PCA (30 PCs), UMAP embedding, and graph-based clustering (resolution 0.5). Cell types were annotated with SingleR using the Human Primary Cell Atlas reference. In GSE169454, nine cell types were identified, with chondrocytes as the predominant population; hub gene expression was detected across the cell landscape (Figure 6A–E). Analysis of GSE220243 yielded a concordant but expanded repertoire of eleven cell types, including additional immune (B cells, monocytes) and endothelial cells (Figure 6F–J). The consistent detection of all six hub genes across both cohorts and diverse cell types confirms their robustness as features of the OA cartilage microenvironment.

### 3.7. Virtual Knockdown Reveals Convergent Perturbation of Inflammatory Pathways

**Figure 7:**
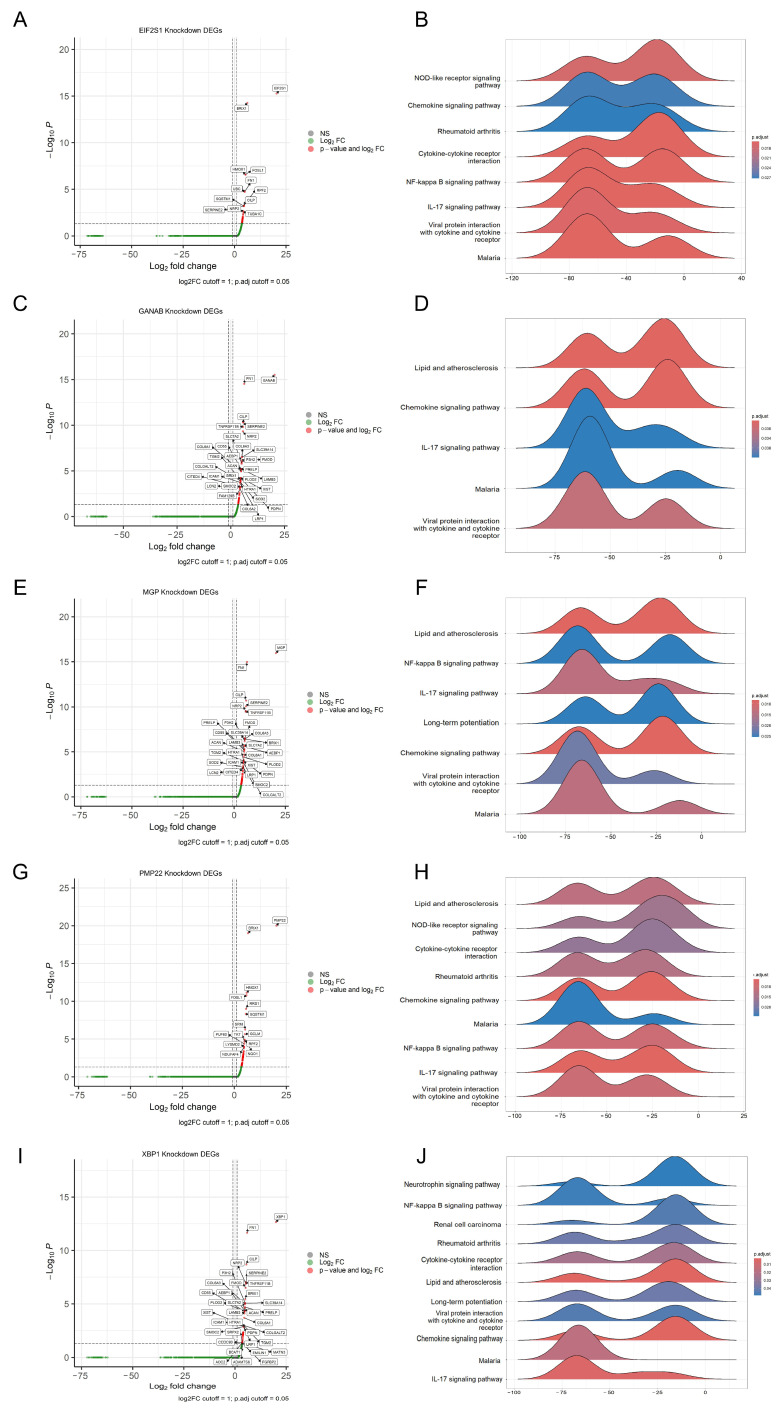
Virtual knockout and pathway enrichment analysis of hub genes in OA chondrocytes (GSE169454). (**A**,**C**,**E**,**G**,**I**) Volcano plots of DEGs upon virtual knockout of EIF2S1, GANAB, MGP, PMP22, and XBP1, respectively. Dashed lines in the volcano plots indicate the significance thresholds: the horizontal dashed line represents the adjusted *p*-value cutoff (p.adj < 0.05), and the vertical dashed lines represent the log_2_ fold change cutoff (|log_2_FC| > 1). (**B**,**D**,**F**,**H**,**J**) GSEA-KEGG enrichment results for the corresponding knockdowns, showing recurrent enrichment of IL-17 signaling, NF-κB signaling, and chemokine signaling pathways (adjusted *p* < 0.1). STT3A knockdown did not yield significant pathway enrichment.

In silico knockout experiments (scTenifoldKnk) in OA chondrocytes were performed to probe the mechanistic roles of the hub genes. Strikingly, five of the six hub genes—EIF2S1, GANAB, MGP, PMP22, and XBP1—converged on a shared core of pro-inflammatory and immune-related signaling cascades. Knockdown of EIF2S1 enriched IL-17 signaling, NF-κB signaling, and NOD-like receptor signaling (Figure 7A,B). GANAB knockdown perturbed IL-17 signaling, chemokine signaling, and lipid/atherosclerosis pathways (Figure 7C,D). MGP knockdown was associated with altered chemokine signaling, IL-17 signaling, and NF-κB signaling (Figure 7E,F). PMP22 knockdown elicited the broadest inflammatory response (Figure 7G,H). XBP1 knockdown resulted in dysregulation of a large panel of inflammatory pathways, including IL-17 and NF-κB signaling (Figure 7I,J). STT3A knockdown did not yield significant pathway enrichment. The recurrent enrichment of IL-17 and NF-κB signaling across five independent knockouts provides strong evidence that these hub genes actively participate in the inflammatory pathophysiology of OA.

## 4. Discussion

In this study, we employed a multi-faceted integrative analytical strategy to dissect molecular dysregulation in OA cartilage, from bulk transcriptomic integrative analysis to single-cell validation and mechanistic inference. Through stringent feature selection, we identified a six-hub-gene signature—EIF2S1, GANAB, STT3A, XBP1, MGP, and PMP22—that exhibits potential diagnostic utility and is computationally inferred to converge on inflammatory signaling pathways, warranting further experimental validation.

Within the scope of our computational framework, the functional annotation of our DEGs prominently highlighted ECM structural components and ER protein processing pathways. This dual enrichment aligns with the unique physiology of chondrocytes as highly secretory cells responsible for maintaining the dense collagenous cartilage matrix [5]. The marked predominance of downregulated genes in OA is consistent with a broad transcriptional repression phenotype, possibly reflecting chondrocyte dedifferentiation, senescence, or the catabolic shift driven by inflammatory cytokines [28]. Four of the six hub genes—EIF2S1, GANAB, STT3A, and XBP1—are intimately involved in ER function and the UPR. EIF2S1 (eIF2α) is the central regulator of the protein kinase R-like endoplasmic reticulum kinase (PERK) branch of the UPR, which attenuates global translation upon ER stress [29]. XBP1, a key transcription factor in the inositol-requiring enzyme 1 alpha (IRE1α) branch, controls the expression of ER chaperones and ER-associated degradation (ERAD) components [30]. GANAB (glucosidase II alpha subunit) and STT3A (catalytic subunit of the oligosaccharyltransferase complex) are critical for protein N-glycosylation and folding quality control in the ER [31]. The persistent selection of these ER-centric genes by LASSO underscores the centrality of ER proteostasis failure in OA chondrocyte dysfunction.

MGP (Matrix Gla Protein) and PMP22 (Peripheral Myelin Protein 22) add additional biological dimensions. MGP is a vitamin K-dependent mineralization inhibitor, and its dysregulation in OA cartilage suggests a contribution to pathological calcification of the articular matrix [32]. PMP22, although classically associated with myelination in peripheral nerves, has emerging roles in cell adhesion, proliferation, and survival [33]. Its strong expression in chondrocytes and prominent inflammatory signature upon knockdown in our study warrant further investigation into its non-canonical function in cartilage.

OA is increasingly recognized as a multifactorial disease driven by complex interactions among multiple cell types, including chondrocytes, synovial fibroblasts, immune cells, and osteocytes, as well as a network of cytokines and growth factors [34,35]. Beyond their individual functions, these six hub genes may operate within an interconnected molecular network in OA chondrocytes. Under ER stress, EIF2S1 and XBP1 coordinate the UPR, while GANAB and STT3A ensure proper N-glycosylation of ECM proteins. Failure of this machinery could lead to secretion of misfolded matrix components and subsequent inflammatory activation. Concurrently, MGP downregulation may predispose the matrix to pathological calcification, which can itself trigger inflammatory responses. PMP22, potentially involved in chondrocyte membrane signaling, may link matrix alterations to intracellular cascades such as NF-κB. The coordinated downregulation of these genes could thus represent a multi-hit mechanism that simultaneously compromises ER proteostasis, ECM quality, and inflammatory restraint. Testing this network hypothesis through co-perturbation experiments would be a logical next step.

A critical aspect of our study is the rigorous distinction between apparent model performance and internally validated generalization. The LOOCV-estimated AUC of 0.769 indicates moderate discrimination, and external validation in independent cohorts is required before any diagnostic application can be considered. This significant gap exemplifies the over-optimism inherent in training-set evaluation and emphasizes the necessity of cross-validation, particularly in biomarker studies with limited sample sizes [36]. The decision curve analysis suggested that the model may provide net benefits over default strategies in this cohort, supporting its preliminary utility for further evaluation.

The molecular subtyping analysis revealed that OA is not a monolithic entity. The C2 subtype, characterized by high UPR and TGF-β signaling activity, may represent an ER stress-driven, pro-fibrotic phenotype, whereas the C1 subtype, with cell cycle and MYC pathway enrichment, suggests a more proliferative or biosynthetic cellular state. This functional divergence provides a framework for precision subtyping, where distinct molecular pathologies may require tailored therapeutic interventions—for instance, targeting the IRE1α/XBP1 pathway in C2 versus cell-cycle regulators in C1. Such a strategy aligns with the broader goal of stratifying OA patients for clinical trials, which historically have failed partly due to patient heterogeneity [3].

The single-cell analysis validated that the hub genes are expressed not only in chondrocytes but also across immune cell populations and stromal cells within the OA joint microenvironment. This broader expression suggests that the hub gene signature may capture pathological signals from a multicellular ecosystem, rather than solely from chondrocytes. The inclusion of immune cells in cartilage samples is consistent with the growing recognition that low-grade inflammation in OA engages multiple cell types [37].

A noteworthy observation from our in silico analysis is the convergent perturbation of inflammatory signaling pathways upon virtual knockout of five distinct hub genes, though these findings remain to be experimentally validated. The recurrent enrichment of IL-17 signaling and NF-κB signaling across five independent knockouts suggests that these hub genes may actively participate in the inflammatory pathophysiology of OA. Their coordinated downregulation in OA could therefore be hypothesized to represent a permissive event that contributes to pathological inflammatory cascades, driving catabolism and disease progression, though direct causal evidence is lacking. IL-17 signaling has been increasingly implicated in OA. Elevated IL-17 levels in synovial fluid and cartilage promote chondrocyte catabolism by upregulating MMPs and ADAMTS-5 while suppressing matrix synthesis [38], consistent with the pathway convergence observed upon virtual knockout of the hub genes. If validated experimentally, the convergence observed here would suggest that the six hub genes function as upstream regulators whose loss of function may contribute to this inflammatory milieu.

Several limitations should be acknowledged. First, the sample size of the integrated training dataset (*n* = 28), although a common challenge in rare tissue transcriptomic studies, is limited and may constrain the generalizability of the diagnostic model; validation in large, independent, and prospectively collected cohorts is essential before any clinical translation. Additionally, several publicly available OA cartilage datasets were not included in the current integration, which may further limit generalizability; future studies incorporating a broader range of cohorts are warranted. Second, the virtual knockout analysis provides computationally inferred mechanistic associations rather than direct causal evidence; these predictions require validation through in vitro gene silencing and biochemical assays in primary chondrocytes or cartilage explants. Third, the bulk RNA-seq datasets used for integration may contain mixed cell populations, and although we incorporated scRNA-seq validation, the relative contribution of each cell type to the bulk signature warrants further deconvolution analysis.

In conclusion, through a comprehensive bioinformatics framework spanning multi-dataset integration, machine learning-based feature selection, molecular subtyping, and single-cell validation, we identified and characterized a six-hub-gene signature for OA cartilage. This signature shows preliminary promise for diagnostic and subtyping applications and reveals a computationally inferred mechanistic convergence on inflammatory signaling pathways, particularly IL-17/NF-κB cascades. These findings provide a hypothesis-generating framework for future functional studies and therapeutic target exploration in osteoarthritis, subject to rigorous experimental validation.

## Data Availability

The datasets analyzed in this study are publicly available. Bulk RNA-seq datasets (GSE285234, GSE287861, GSE289464) and single-cell RNA-seq datasets (GSE169454, GSE220243) were downloaded from the Gene Expression Omnibus (GEO). All data used are publicly accessible; further inquiries can be directed to the corresponding author.

## Supplementary Materials

The following supporting information can be downloaded at https://www.mdpi.com/article/10.3390/genes17060696/s1: Supplementary Table S1: The list of DEG; Supplementary Table S2: The list of GEO datasets.
